# Inhibition of Mitochondrial Complex I Leads to Decreased Motility and Membrane Integrity Related to Increased Hydrogen Peroxide and Reduced ATP Production, while the Inhibition of Glycolysis Has Less Impact on Sperm Motility

**DOI:** 10.1371/journal.pone.0138777

**Published:** 2015-09-25

**Authors:** María Plaza Davila, Patricia Martin Muñoz, Jose A. Tapia, Cristina Ortega Ferrusola, Carolina Balao da Silva C, Fernando J. Peña

**Affiliations:** 1 Laboratory of Equine Reproduction and Equine Spermatology, Veterinary Teaching Hospital, University of Extremadura, Cáceres, Spain; 2 Department of Physiology, Faculty of Veterinary Medicine, University of Extremadura, Cáceres, Spain; Clermont-Ferrand Univ., FRANCE

## Abstract

Mitochondria have been proposed as the major source of reactive oxygen species in somatic cells and human spermatozoa. However, no data regarding the role of mitochondrial ROS production in stallion spermatozoa are available. To shed light on the role of the mitochondrial electron transport chain in the origin of oxidative stress in stallion spermatozoa, specific inhibitors of complex I (rotenone) and III (antimycin-A) were used. Ejaculates from seven Andalusian stallions were collected and incubated in BWW media at 37°C in the presence of rotenone, antimycin-A or control vehicle. Incubation in the presence of these inhibitors reduced sperm motility and velocity (CASA analysis) (p<0.01), but the effect was more evident in the presence of rotenone (a complex I inhibitor). These inhibitors also decreased ATP content. The inhibition of complexes I and III decreased the production of reactive oxygen species (p<0.01) as assessed by flow cytometry after staining with CellRox deep red. This observation suggests that the CellRox probe mainly identifies superoxide and that superoxide production may reflect intense mitochondrial activity rather than oxidative stress. The inhibition of complex I resulted in increased hydrogen peroxide production (p<0.01). The inhibition of glycolysis resulted in reduced sperm velocities (p<0.01) without an effect on the percentage of total motile sperm. Weak and moderate (but statistically significant) positive correlations were observed between sperm motility, velocity and membrane integrity and the production of reactive oxygen species. These results indicate that stallion sperm rely heavily on oxidative phosphorylation (OXPHOS) for the production of ATP for motility but also require glycolysis to maintain high velocities. These data also indicate that increased hydrogen peroxide originating in the mitochondria is a mechanism involved in stallion sperm senescence.

## Introduction

The mitochondria of the spermatozoa control numerous functions and are considered to be hallmarks of sperm functionality [1, 2]. In addition to their role as an ATP source via oxidative phosphorylation (OXPHOS), other functions in regulating the lifespan of spermatozoa have attracted major research attention to these organelles [1] [3]. Important cellular functions in the spermatozoa are redox-regulated; the production of reactive oxygen species (ROS) is an early event during the series of the modifications that occur during capacitation[4]. However alteration in the redox homeostasis of the cell leads to sperm senescence and finally death[5]; in humans, it has been reported that the mitochondria of defective sperm are the major source of ROS originating from electron leakage in the electron transport chain (ETC) [6]. This has also been assumed to be true for horses, as reviewed in [7], and recent data support this hypothesis[8]. Moreover, sperm biotechnologies that are widely used in animal breeding are known to alter the redox status of these cells [9]. The preservation of spermatozoa for short periods in a liquid state or frozen for longer periods is at the core of the horse industry, so the investigation of mechanisms leading to sperm senescence, infertility and finally death, has increased in the recent years. Apoptotic-like mechanisms [10–13] are responsible for sperm deterioration during conservation. One mechanism explaining sperm senescence (and ultimately sperm death) during storage is lipid peroxidation [14]. This is related to the highly unsaturated nature of the lipids that comprise sperm membranes, which predisposes them to oxidative attack [15]. Interestingly the mitochondria are considered to be more sensitive to the changes induced by cryopreservation and cooling than other organelles in spermatozoa [16, 17]. The disruption of the mitochondrial electron transport chain is known to induce the production of mitochondrial ROS both in somatic cells [18] and human sperm [6]. To specifically assess the situation in stallion and test the hypothesis that the disruption of complexes I and III leads to sperm malfunction due to oxidative stress, split samples were incubated in presence of rotenone and antimycin-A. The production of reactive oxygen species was monitored using flow cytometry. Other effects on sperm parameters were investigated as well, moreover the role of glycolysis in sperm function was investigated using specific inhibitors and through the incubation of stallion spermatozoa in media free of glucose and pyruvate.

## Materials and Methods

### Reagents and media

Ethidium homodimer, 5,5’,6,6’–tetrachloro-1,1’,3,3’ tetraethylbenzymidazolyl carbocianyne iodine (JC-1), YO-PRO-1, CellRox Deep Red Reagent, Hoechst 33342, hydroethidine, dichlorodihydrofluoresceindiacetate, Mitosox, Sytox green and the ATP detection Kit were from Molecular Probes (Molecular Probes, Leiden The Netherlands). Rotenone, antimycin-A, 2-Deoxy-D-glucose and all other chemicals were from Sigma (St Louis MO)

### Semen collection and processing

The use of animals for semen collection was approved by the ethics committee on animal experimentation of the University of Extremadura with authorization number 103/2013. Semen was obtained from seven Pure Spanish horses (PRE) (three ejaculates each from animals that were 6 to 14 years old and of proven fertility due to regular use as stud stallions in our center) that were individually housed at the Veterinary Teaching Hospital of the University of Extremadura, Cáceres, Spain. The stallions were maintained according to institutional and European regulations, and the sperm samples were collected on a regular basis (two collections/week) during the 2013 and 2014 breeding seasons. Ejaculates were collected by staff veterinarians, using a pre-warmed, lubricated Missouri model artificial vagina with an inline filter to eliminate the gel fraction. The semen was immediately transported to the laboratory for evaluation and processing. The ejaculate was extended 1:2 in INRA-96 (IMV l’Aigle, France), centrifuged (600 x g for 10 min), and re-suspended in BWW media (91.06 mM NaCl, 4.78 mM KCl, 2.44 mM MgSO4, 1.17 mM KPO4, 21.0 mM HEPES, 5.5 mM glucose [anhydrous], 0.25 mM sodium pyruvate, 1.71 mM lactic acid hemicalcium salt, and 21.55 mM sodium lactate supplemented with 1% PVA at 300 mOsm/kg). The pH of the suspension was adjusted to 7.2. All media were filtered through a 0.45-mm filter. The final concentration for sperm incubation was 40 x10^6^ spmtz/ml.

All experiments followed a split sample design, with every ejaculate divided to create control and treatment groups; for this, the sperm aliquots were incubated at 37° C in presence of the inhibitors or controls (vehicle) and aliquots were taken after 60, 120, and 180 min for CASA, flow cytometry and ATP measurements.

### Sperm motility

Sperm motility and kinematics were assessed by analyzing undiluted aliquots taken at 60, 90 and 180 minutes of incubation with a CASA system (ISAS® Proiser, Valencia, Spain) and 20 μm deep Leja chambers (Leja Products B.V. Nieuw Vennep, The Netherlands) placed on a warmed (37°C) stage. A minimum of 500 spermatozoa per sample were analyzed in four different randomly selected fields. The analysis was based on the examination of 60 consecutive digital images in a lapse time of one second (60 Hz) obtained using a 10x negative phase contrast objective. The number of objects incorrectly identified as spermatozoa was minimized on the monitor using the playback function. With respect to the setting parameters for the program, spermatozoa with a VAP <15 μm/s were considered to be immotile, whereas spermatozoa with a velocity >15 μm/s were considered to be motile. Spermatozoa deviating <45% from a straight line were designated as linearly motile, and spermatozoa with a curvilinear velocity (VCL) > 45 μm/s were designated as rapid sperm. The absolute and re-calculated kinematic parameters measured by CASA included the following: curvilinear velocity (VCL; μm/s), which measures the sequential progression along the true trajectory; straight line velocity (VSL; μm/s), which measures the straight trajectory of the spermatozoa per unit time; and average path velocity (VAP; μm/s), which measures the mean trajectory of the spermatozoa per unit of time.

### Flow cytometry

Flow cytometry analyses were conducted using a MACSQuant Analyser 10 (Miltenyi Biotech) flow cytometer equipped with three lasers emitting at 405 nm, 488 nm, and 635 nm and 10 photomultiplier tubes (PMTs) (V1 (excitation 405 nm, emission 450/50 nm), V2 (excitation 405 nm, emission 525/50 nm), B1 (excitation 488 nm, emission 525/50 nm), B2 (excitation 488 nm, emission 585/40 nm), B3 (excitation 488 nm, emission 655–730 nm (655LP + split 730), B4 (excitation 499 nm, emission 750 LP), R1 (excitation 635 nm, emission 655–730 nm (655LP+split 730) and R2 (excitation 635 nm, emission filter 750 LP). The system was controlled using MACSQuantify software. Sperm subpopulations were divided by quadrants to quantify the frequency of each subpopulation. Forward and sideways light scatter were recorded for a total of 50,000 events per sample. Non-sperm events were eliminated by gating the sperm population after Hoechst 33342 staining. The instrument was calibrated daily using specific calibration beads provided by the manufacturer, and compensation overlap was performed before each experiment using appropriate unstained and single-stained controls.

#### Simultaneous flow cytometric assessment of subtle membrane changes, viability and oxidative stress (reactive oxygen species ROS)

**Fig 1:**
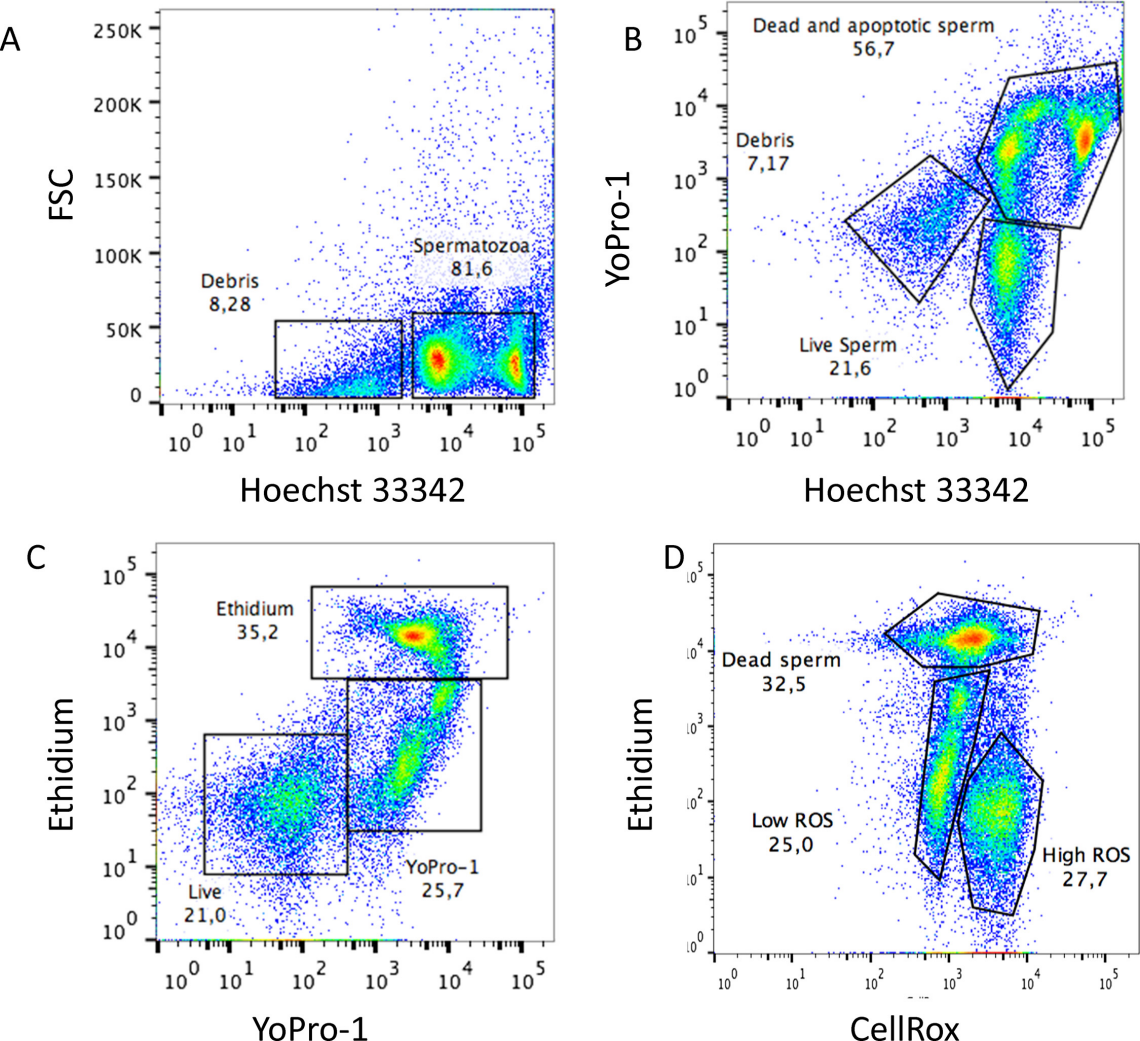
Representative cytograms for simultaneous flow cytometric assessment of subtle membrane changes, viability and reactive oxygen species (ROS). Samples were stained with Hoechst 33342, Yo-Pro-1, ethidium homodimer (Eth) and CellROX Deep Red, as described in the ‘Materials and methods’ section. Hierarchical gating was applied to exclude debris from the analysis and simultaneously measure viability, apoptosis, necrosis and ROS. (A) Hoechst 33342 fluorescence was detected using the V1 channel (Ex 405 bandpass filter 450/50 nm), and a gate was applied to positive (DNA-containing particles) events to gate out debris. The gated region was analysed. (B) Yo-Pro-1 was detected using the B1 channel (Ex 488 bandpass Filter 525/50 nm) and Hoechst 33342 fluorescence was detected using the V1 channel (Ex 405 bandpass filter 450/50 nm) (C,) Yo-Pro-1 was detected using the B1 channel (Ex 488 bandpass Filter 525/50 nm) and Eth was detected using the B3 channel (Ex 488 bandpass filter 655–730 nm) and (D) CellROX Deep Red was detected using the R1 channel (Ex 635 nm bandpass filter 655–730 nm). Positive controls for oxidation and compromised membranes are presented obtained as describe in material and methods.

Aliquots were taken at specific time intervals and diluted in PBS for staining to a final concentration 5 x 10^6^ spermatozoa/mL. Aliquots were stained with CellRox (5μM) and Hoechst 33342 (0.5μM). After thorough mixing, the sperm suspension was incubated at RT in the dark for 25 min. The spermatozoa were then washed with PBS, then ethidium homodimer (0.35μM) and Yo-Pro-1 (25nM) were added, and the mixture was incubated for five minutes and read in a flow cytometer. This staining protocol was a modified version [19] of previously published protocols [20–22] and distinguishes four sperm subpopulations while simultaneously measuring oxidative stress. The first subpopulation, positive for only Hoechst 33342, was considered to be alive and without any membrane alterations. Another subpopulation, the Yo-Pro-1-positive cells emitting green fluorescence, represents cells whose membranes have become slightly permeable, enabling Yo-Pro-1 but not ethidium homodimer to cross the plasma membrane. Finally, two subpopulations of dead spermatozoa were easily detected; spermatozoa staining both with Yo-Pro-1 and ethidium homodimer (emitting both green and red fluorescence) and cells staining with only ethidium homodimer, emitting red fluorescence. Spermatozoa exhibiting oxidative stress emit fluorescence in the far red spectrum, whereas Hoechst 33342-positive sperm emit blue fluorescence. The positive controls for oxidative stress were samples supplemented with 800 μM SO_4_Fe and 1.7 M of H_2_O_2_ (Sigma) to induce the Fenton reaction. Representative dot plots of this assay depicting positive controls are present in Fig 1.

#### Evaluation of mitochondrial membrane potential (ΔΨm)

The probe 5,5’,6,6’–tetrachloro-1,1’,3,3’ tetraethylbenzymidazolyl carbocianyne iodine (JC-1), forms multimeric aggregates in mitochondria with high membrane potential; these aggregates emit in the high orange wavelength of 590 nm when excited at 488 nm. In mitochondria with low membrane potential, JC-1 forms monomers that emit in the green wavelength (525 to 530 nm) when excited at 488 nm. Published staining protocols from our laboratory and others were followed [16, 23]. Briefly each sperm sample,was stained with JC-1 (1.5μM) were incubated at 37°C in the dark for 40 min before flow cytometry analysis.

#### Determination of anion superoxide (O2•-) and hydrogen peroxide (H2O2) production

Following protocols that have been previously validated for stallion sperm [24, 25], the samples were stained with hydroethidine (1.4μM) to detect superoxide anion (O_2_•-) and dichlorodihydrofluorescein diacetate (H_2_DCFDA 5 μM) to detect H_2_0_2_. To restrict our analysis to spermatozoa Hoechst 33342 (0.5μM) was added. The samples were incubated for 30 min at 38°C before analyzing 100.000 events in the flow cytometer. HE and H_2_DCFDA were excited at 488 nm, and fluorescence was recorded at 530 and 610 nm, respectively. Hoechst 33342 was excited at 405 nm, and fluorescence was recorded at 450 nm.

#### Mitosox red assay

The generation of mitochondrial superoxide anion was investigated using Mitosox Red (MSR, Molecular probes) as previously described [26, 27] with slight adaptations for stallion sperm in our laboratory[19]. Spermatozoa (5 × 10^6^ /mL) were stained with 2 μM MitoSox Red, incubated for 15 min at 37° C, centrifuged for 5 min at 600 × *g*, and resuspended in BWW. SYTOX Green (0.05 μM) was then added for a final 15 min incubation. The MSR (red) and SYTOX Green (green) fluorescence were then measured using 530/30 band pass (green) and 585/42 band pass (red) filters. Non sperm-specific events were gated out after staining with Hoechst 33342 (0.5μM), and 10,000 cells were examined.

### Determination of intracellular ATP

Intracellular ATP content in sperm lysates was measured using the ATP determination Kit (A22066) (Molecular Probes, Leiden Holland) according to the instructions provided by the manufacturer and previously published protocols from our laboratory and others [8, 28]. Aliquots of spermatozoa (200 μL) were snap frozen in liquid nitrogen and stored at -80°C until analysis. On the day of analysis, the samples were thawed on ice and centrifuged at 20,000 x g for 15 min at 4° C, and the supernatant was used for the assay. The assay is based on the luciferase’s requirement for ATP in producing light (emission maxima at approximately 560 nm at pH 7.8). The ATP content was normalized to pM/100 μg of protein quantified colorimetrically [29].

### Statistical analysis

Three ejaculates from 7 individual stallions were collected. All experiments were repeated at least 21 times (three ejaculates from each of the seven stallions). The normality of the data was previously assessed using the Kolmogorov-Smirnoff test. In light of the normality of the distribution, results were analyzed by ANOVA, and Dunnett´s t-test was used to perform comparisons. The Pearson test was used to study the correlations, in control samples, between the production of reactive oxygen species and parameters of sperm functionality. Power analysis was set at 0.8 and P < 0.05 was regarded as significant, with * p<0. 05 and ** p<0.01. The results are provided as the means ± SD. All analyses were conducted using SPSS 19.0 software for Mac.

## Results

### Inhibition of complex I (NADH-ubiquinone oxidoreductase) and complex III (co-enzyme Q-cytrochrome c reductase) reduces stallion sperm motility

**Fig 2:**
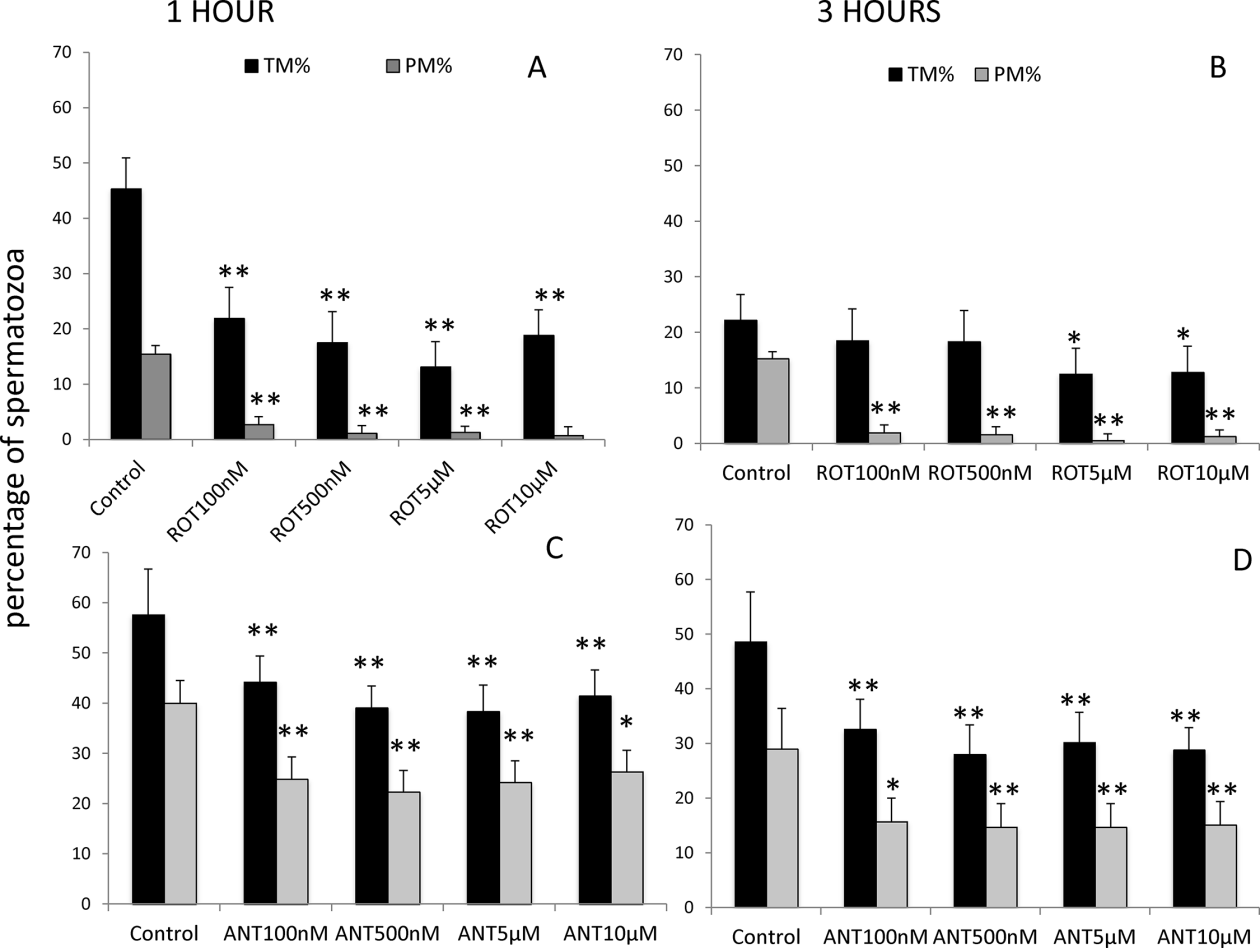
Effect of the inhibition of Complex I of the electron transport chain on stallion sperm motility (CASA analysis). Stallion spermatozoa were extended in BWW media and incubated in presence of rotenone (0 nm (DMSO), 100 nm, 500 nm, 5 μM and 10 μM) and antimycin-A (0 vehicle DMSO, 100 nm, 500 nm, 5 μM and 10 μM) as indicated in the materials and methods section; motility was evaluated after 1 and three hours of incubation. TM%—percentage of total motile spermatozoa, PM %—percentage of progressive motile spermatozoa. Comparisons are made against controls. * P<0.05, ** P<0.01. The results are given as the means ± SD. The graphics represent two independent experiments (one for antimycin-A and one for rotenone) following a split sample design (the same ejaculate was split into the treatment and control groups).

Stallion sperm were washed and extended in BWW media supplemented with increasing concentrations of the mitochondrial complex I inhibitor rotenone (0 (vehicle) 100 and 500 nM and 5 and 10 μM). After 1 and 3 h of incubation at 37° C, motility was determined using a CASA system. After one hour of incubation, all the concentrations of the inhibitors tested reduced the percentage of motile spermatozoa, whereas after 3 hours of incubation, total motility was reduced in samples incubated in presence of concentrations of rotenone over 5 μM. Progressive motility dropped with all the concentrations tested after 1 and 3 hours of incubation at 37°C (Fig 2A and 2B).

Incubation of stallion spermatozoa in presence of the mitochondrial complex III inhibitor resulted in decreased total and progressive motilities after 1 and three hours of incubation at 37°C in BWW media at all the concentrations tested (Fig 2C and 2D).

### Inhibition of complex I (NADH-ubiquinone oxidoreductase) and complex III (co-enzyme Q-cytrochrome c reductase) reduces stallion sperm velocities

**Fig 3:**
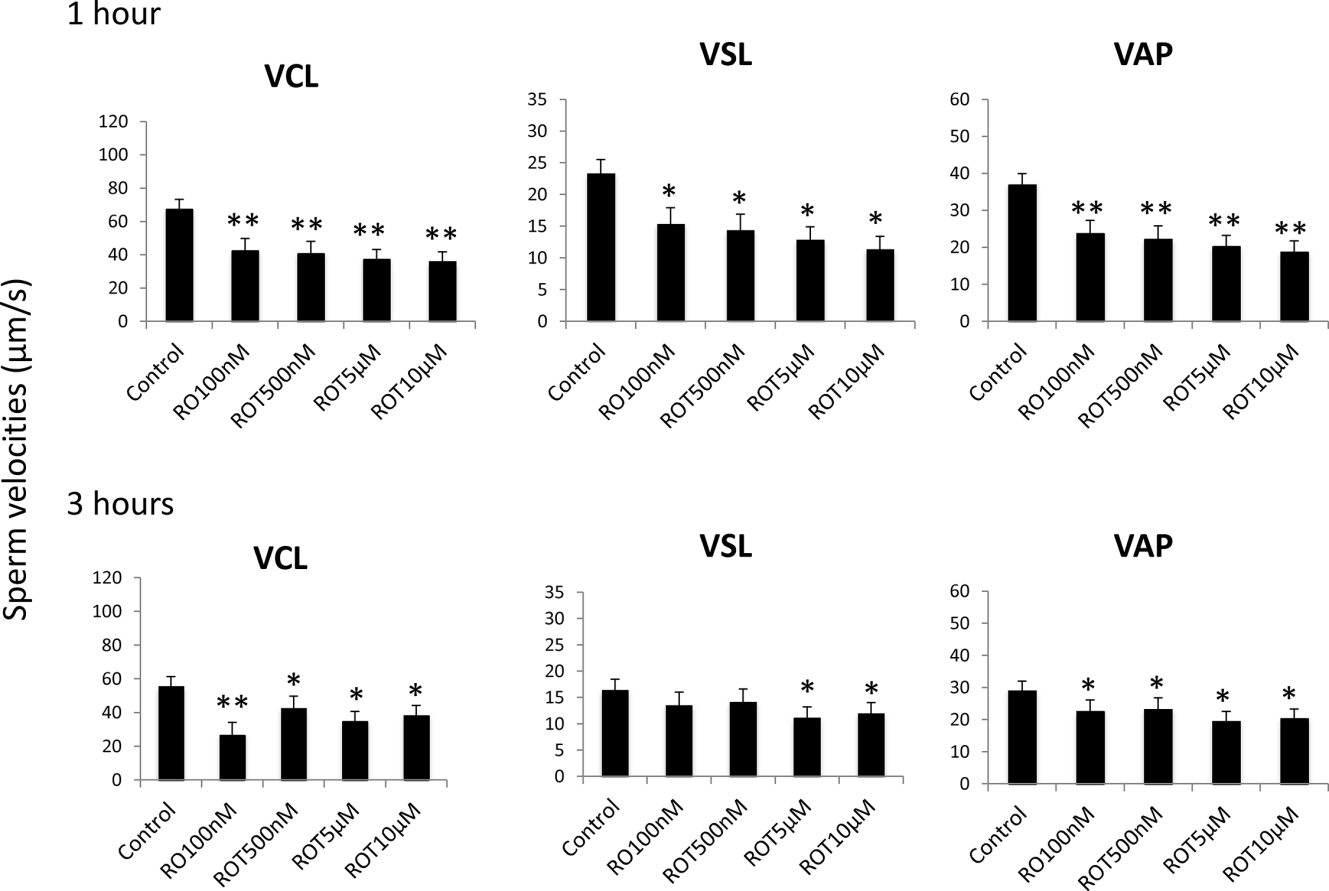
Effect of the inhibition of Complex I of the electron transport chain on stallion sperm velocities (CASA analysis). Stallion spermatozoa were extended in BWW media and incubated in the presence of rotenone (0 nm (DMSO), 100 nm, 500 nm, 5 μM and 10 μM) as indicated in the materials and methods section; velocities were evaluated after 1 and three hours of incubation, VCL—curvilinear velocity (μM/s), VSL straight line velocity (μM/), VAP average path velocity (μM/s). * P<0.05, ** P<0.01. The results are given as the means ± SD.

**Fig 4:**
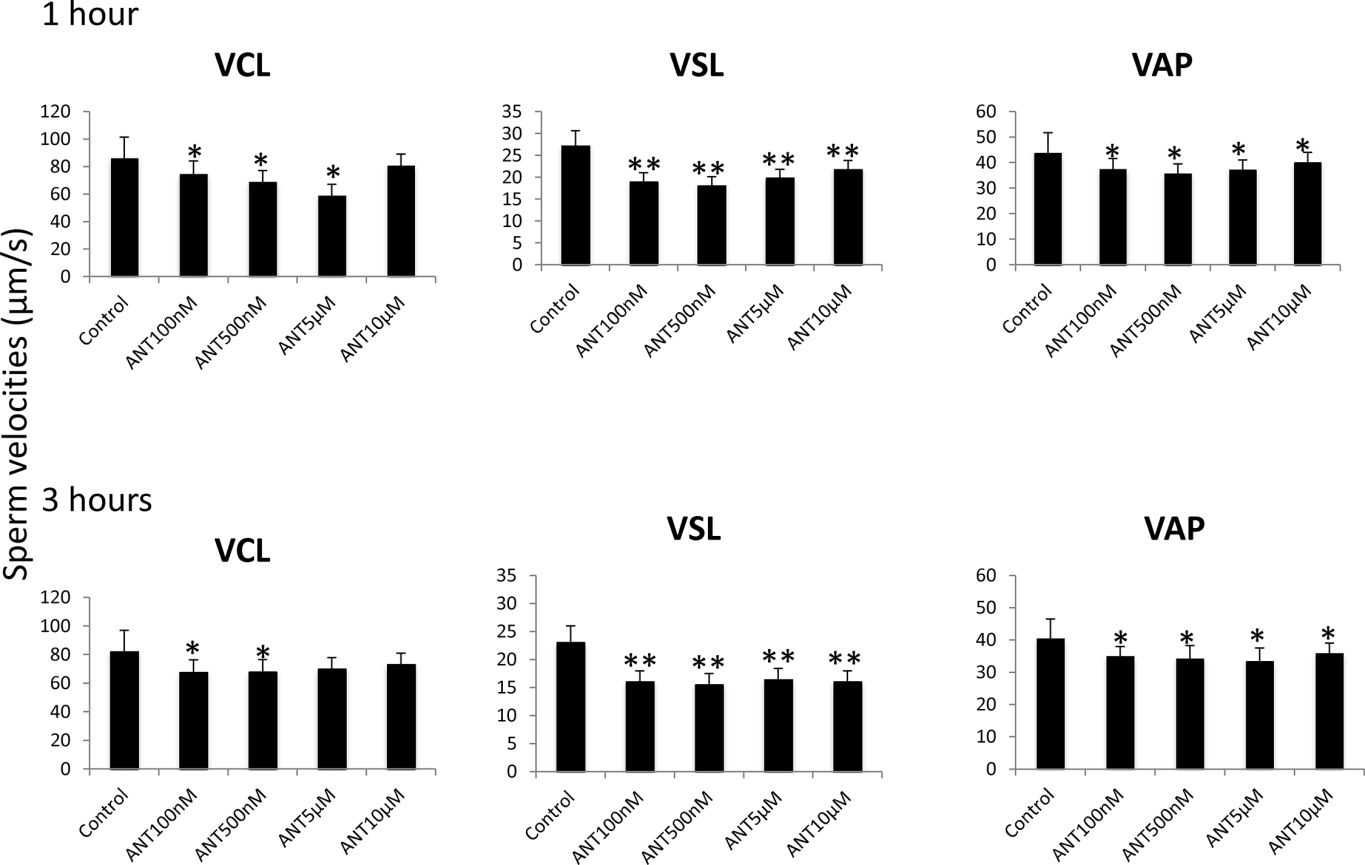
Effect of the inhibition of Complex III of the electron transport chain on stallion sperm velocities (CASA analysis). Stallion spermatozoa were extended in BWW media and incubated in the presence of antimycin-A (0 vehicle DMSO, 100 nm, 500 nm, 5 μM and 10 μM) as indicated in the materials and methods; velocities were evaluated after 1 and three hours of incubation, VCL—curvilinear velocity (μM/s), VSL—straight-line velocity (μM/s), VAP—average path velocity (μM/s). * P<0.05, ** P<0.01. The results are given as the means ± SD.

To determine the effect of mitochondrial complex I inhibition on sperm velocities, split samples were incubated in presence of rotenone, and sperm velocities were determined after one and three hours of incubation using a CASA system. All the concentrations tested reduced circular, straight line, and average velocities after one hour of incubation. After three hours of incubation, VCL and VAP followed the same tendency, but VSL was reduced only in the presence of concentrations of rotenone greater than 5 μM (Fig 3). The mitochondrial complex III inhibitor antimycin- A reduced circular velocity at all concentrations tested after one hour of incubation and at concentrations of 100 nM and 500 nM after three hours of incubation. Straight-line and average path velocities were reduced by all the concentrations tested and after one and three hours of incubation (Fig 4).

### Inhibition of complex I (NADH-ubiquinone oxidoreductase) and complex III (co-enzyme Q-cytochrome c reductase) reduces the percentage of spermatozoa with intact membranes

**Fig 5:**
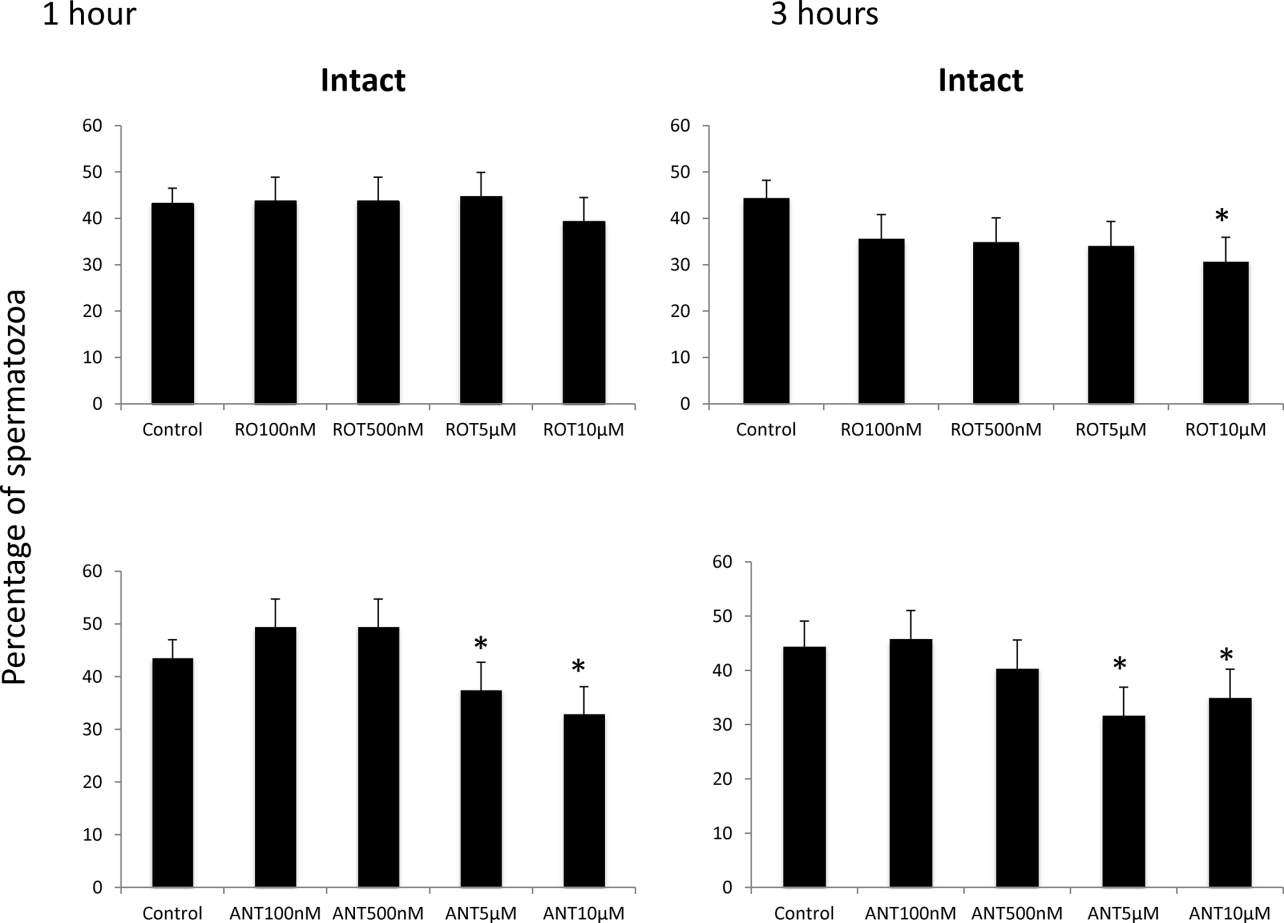
Effects of the inhibition of Complexes I and III of the electron transport chain on the percentage of stallion spermatozoa showing intact membranes. Stallion spermatozoa were extended in BWW media and incubated in the presence of rotenone (0 nm (DMSO), 100 nm, 500 nm, 5 μM and 10 μM) or antimycin-a (0 (DMSO), 100 nm, 500 nm, 5 μM and 10 μM) as indicated in the materials and methods; membrane integrity was determined using the H33342/YoPr/Ethidium assay as described in the materials and methods. ** P<0.01. The results are given as the means ± SD.

Rotenone had no effect on the percentage of intact sperm membranes after 1 hour of incubation, but after three hours of incubation, 10 μM rotenone reduced the percentage of intact sperm. On the other hand, the inhibitor of complex III reduced the percentages of intact sperm (p<0.05) after 1 and three hours of incubation at concentrations of 5 and 10 μM (Fig 5).

### Effect of complex I (NADH-ubiquinone oxidoreductase) and complex III (co-enzyme Q-cytochrome c reductase) on reactive oxygen species (ROS) production by stallion spermatozoa

**Fig 6:**
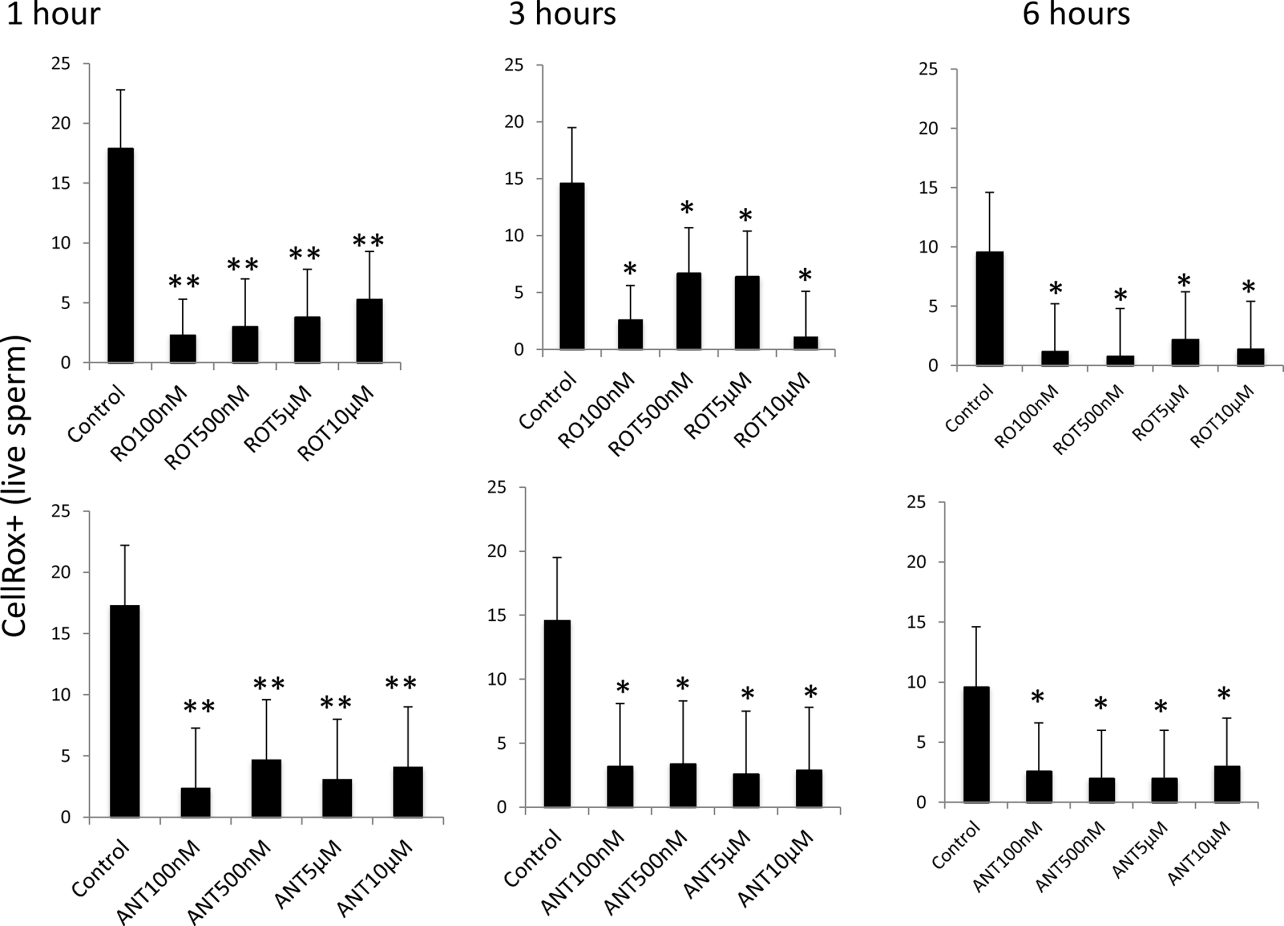
Effect of the inhibition of Complexes I and III of the electron transport chain on the percentage of stallion spermatozoa showing ROS production using CellRox deep red reagent. Stallion spermatozoa were extended in BWW media and incubated in the presence of rotenone (0 (DMSO), 100 nm, 500 nm, 5 μM and 10 μM) or antimycin (0 (DMSO), 100 nm, 500 nm, 5 μM and 10 μM) as indicated in the materials and methods. ROS were measured using flow cytometry after CellRox deep red staining as described in the materials and methods. * p<0.05, ** p<0.01. The results are given as the means ± SD.

**Fig 7:**
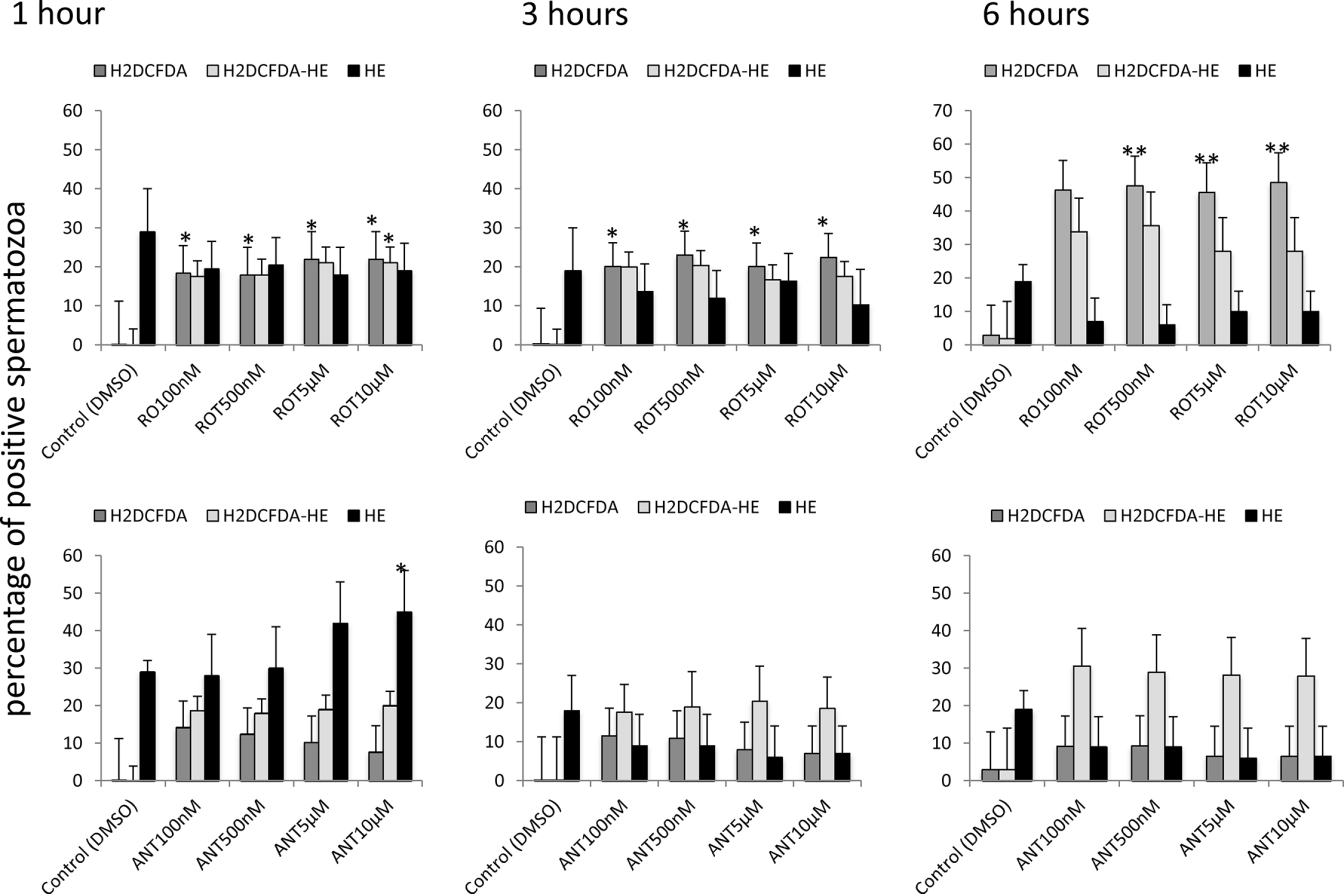
Effect of the inhibition of Complexes I and III of the electron transport chain on superoxide and hydrogen peroxide production by stallion spermatozoa. Stallion spermatozoa were extended in BWW media and incubated in the presence of rotenone or antimycin as indicated in the materials and methods. Superoxide and hydrogen peroxide were measured using flow cytometry after HE (superoxide) and H_2_DCFDA (hydrogen peroxide) staining as described in the materials and methods. * p<0.05, ** p<0.01. The results are given as the means ± SD.

**Fig 8:**
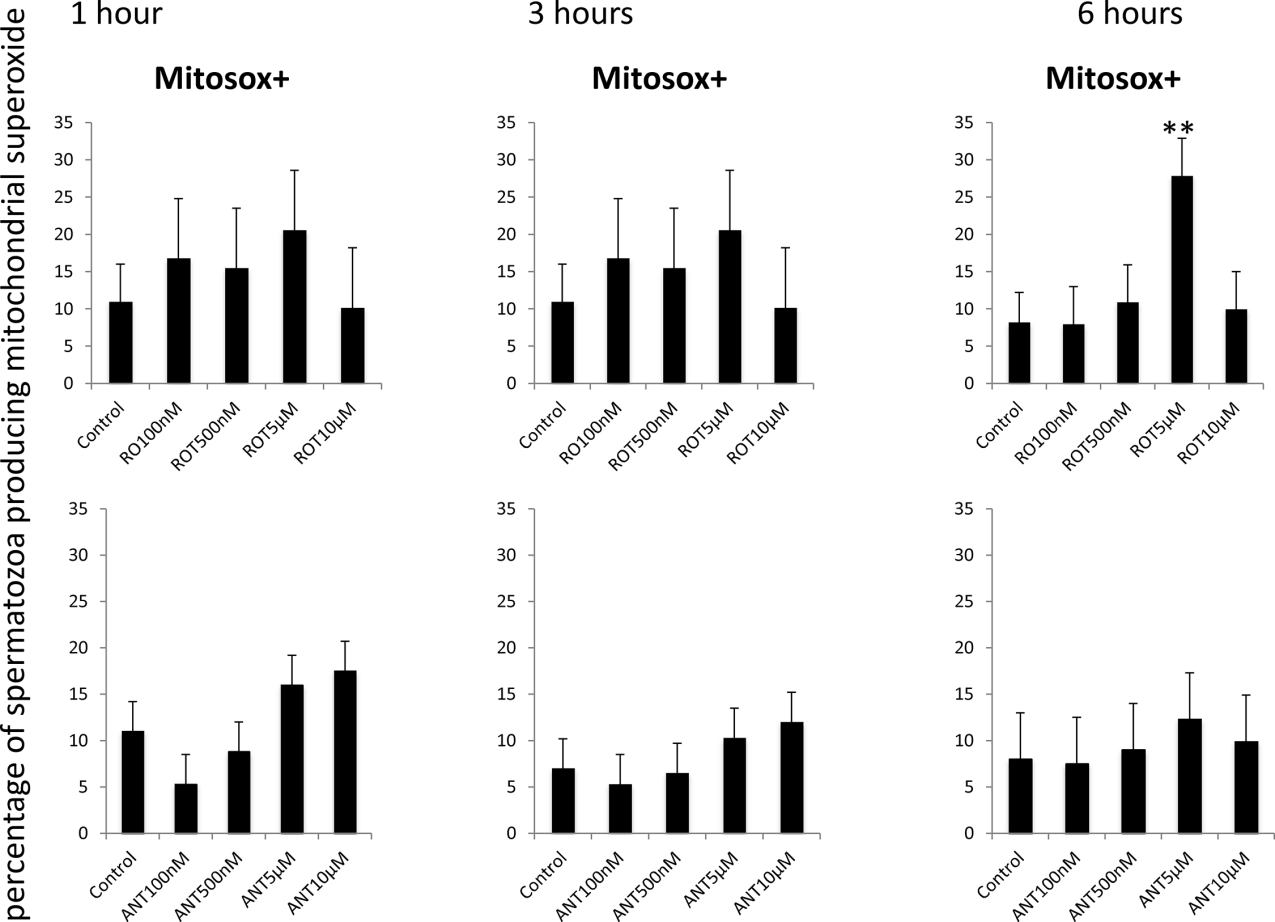
Effect of the inhibition of Complexes I and III of the electron transport chain on the production of mitochondrial superoxide in stallion spermatozoa. Stallion spermatozoa were extended in BWW media and incubated in the presence of rotenone or antimycin as indicated in the materials and methods. Mitochondrial superoxide was measured using flow cytometry after Mitosox staining as described in the materials and methods. Data represent Mitosox positive sperm in the whole population. * p<0.05, ** p<0.01. The results are given as the means ± SD.

The production of reactive oxygen species was initially monitored using CellRox using previously published protocols from our laboratory [19]. Both inhibitors significantly decreased ROS production at all incubation times (Fig 6). In light of these paradoxical results, reactive oxygen species production was also monitored using specific probes for superoxide anion, hydrogen peroxide and mitochondrial superoxide anion. Rotenone increased hydrogen peroxide production during incubation at 37°C, with no changes in non-mitochondrial superoxide anion (Fig 7). Following a similar pattern, rotenone induced mitochondrial superoxide production after 6 hours of incubation at 37°C (Fig 8). The inhibition of complex III with antimycin resulted in increased superoxide production only after one hour of incubation.

### Inhibition of complex I (NADH-ubiquinone oxidoreductase) and complex III (co-enzyme Q-cytochrome c reductase) induces mitochondrial uncoupling and reduces ATP content in stallion spermatozoa

**Fig 9:**
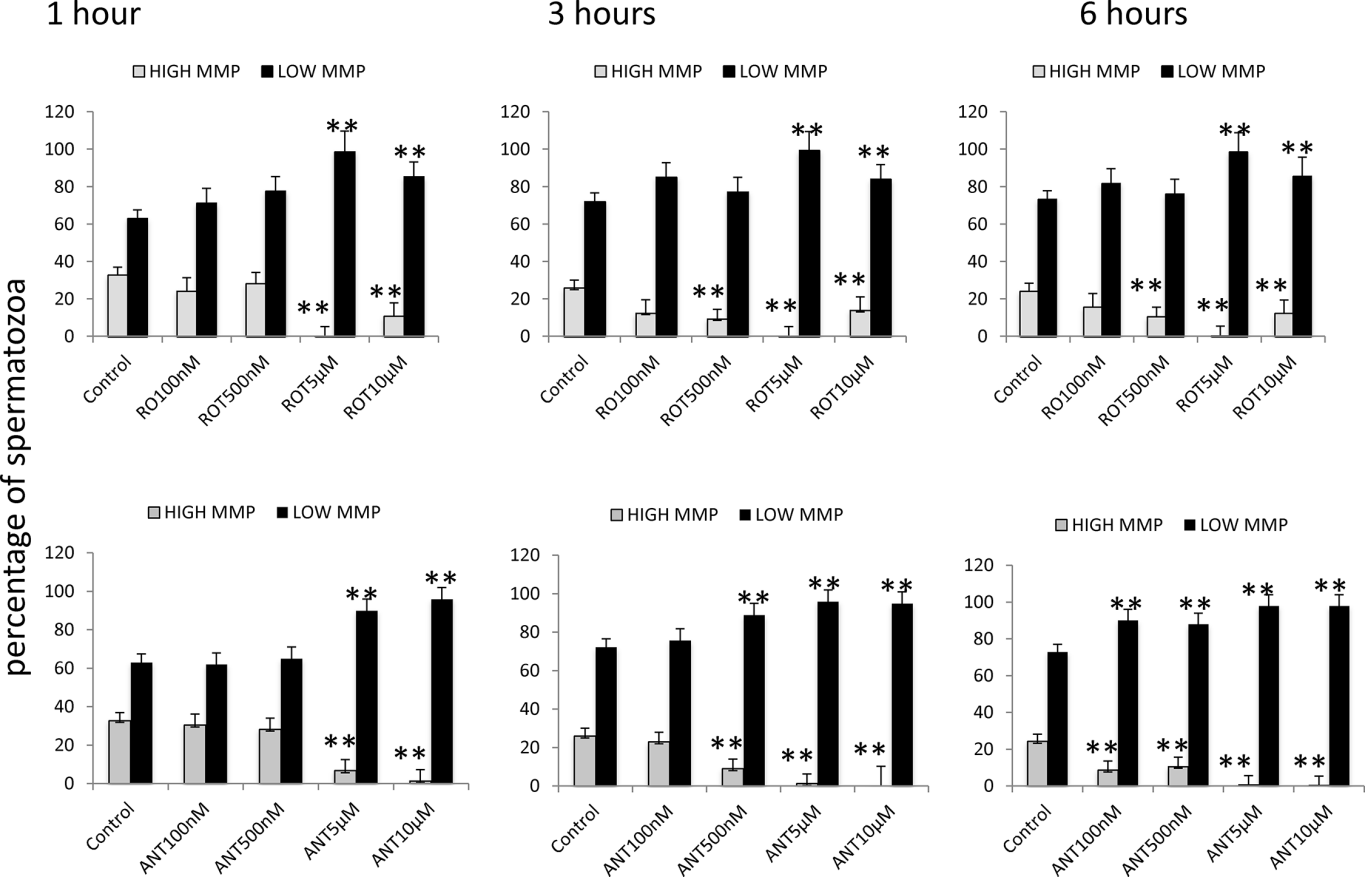
Effects of the inhibition of Complexes I and III of the electron transport chain on the mitochondrial membrane potential of stallion spermatozoa. Stallion spermatozoa were extended in BWW media and incubated in the presence of rotenone (0 (DMSO), 100 nm, 500 nm, 5 μM and 10 μM) or antimycin-A (0 (DMSO), 100 nm, 500 nm, 5 μM and 10 μM) as indicated in the materials and methods. Mitochondrial membrane potential was assessed using flow cytometry after JC-1 staining. High MMP, spermatozoa showing high mitochondrial membrane potential, Low MMP, spermatozoa showing low mitochondrial membrane potential ** P<0.01. The results are given as the means ± SD.

**Fig 10:**
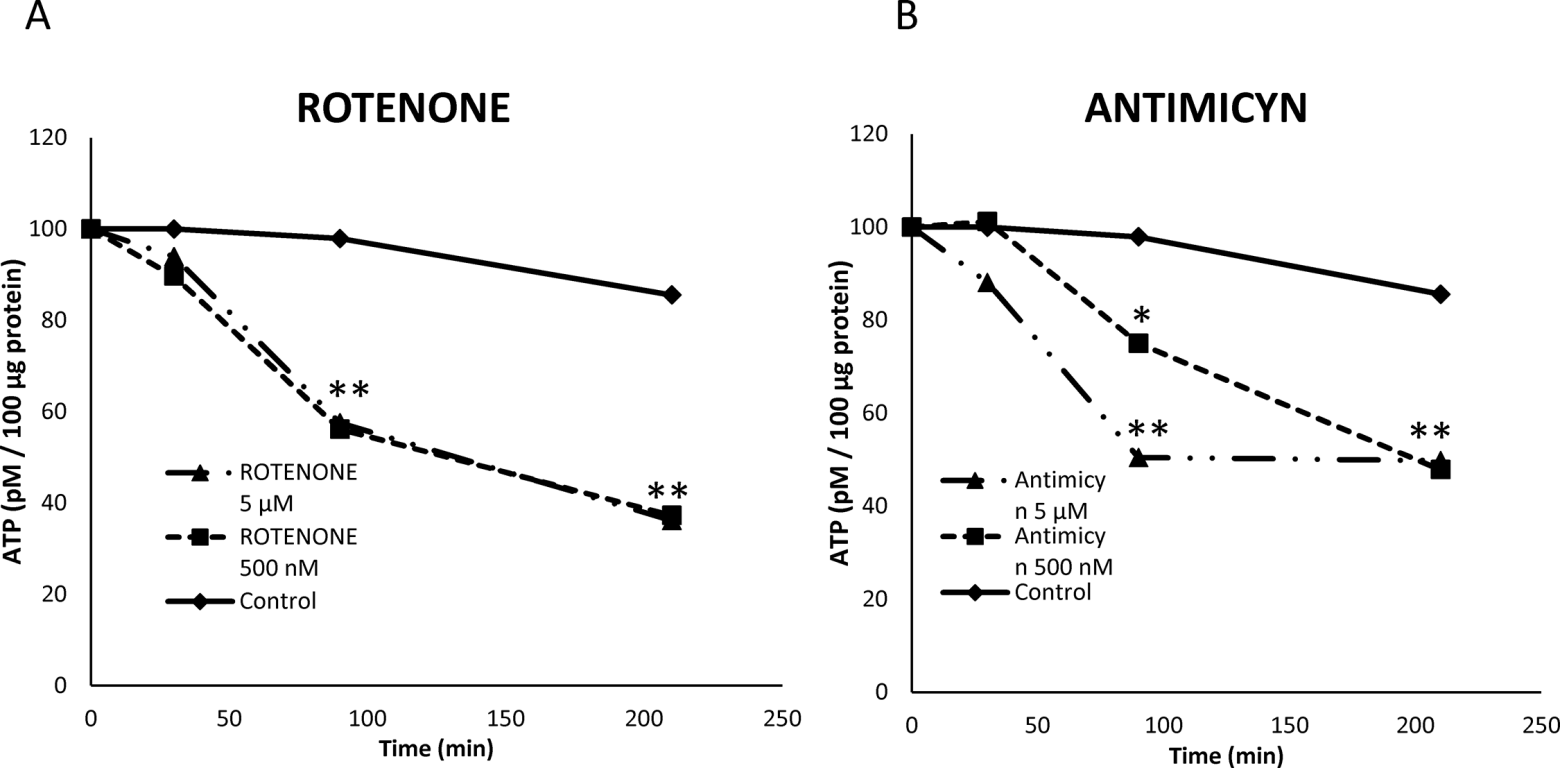
Effect of the inhibition of Complexes I and III of the electron transport chain on the ATP content of stallion spermatozoa. Stallion spermatozoa were extended in BWW media and incubated in the presence of rotenone (0 (DMSO), 100 nm, 500 nm, 5 μM and 10 μM) or antimycin (0 (DMSO), 100 nm, 500 nm, 5 μM and 10 μM) as indicated in the materials and methods. ATP was determined as described in the materials and methods * p<0.05, ** p<0.01. The results are given as the means ± SD.

One possible explanation for reduction of sperm motility and velocities is reduced ATP production due to mitochondrial uncoupling. To evaluate this possibility mitochondrial membrane potential was evaluated using JC-1 and ATP content using a luciferin-luciferase based assay. Inhibition of both complexes I and III lead to mitochondrial uncoupling when rotenone and antimycin were present at concentrations over 5 μM (Fig 9). The dynamics of ATP production was assessed in samples incubated in presence of both inhibitors up to 250 minutes. Both inhibitors reduced ATP content in stallion spermatozoa after 100 minutes of incubation (Fig 10).

### Effect of inhibition of glycolysis on stallion sperm function

**Fig 11:**
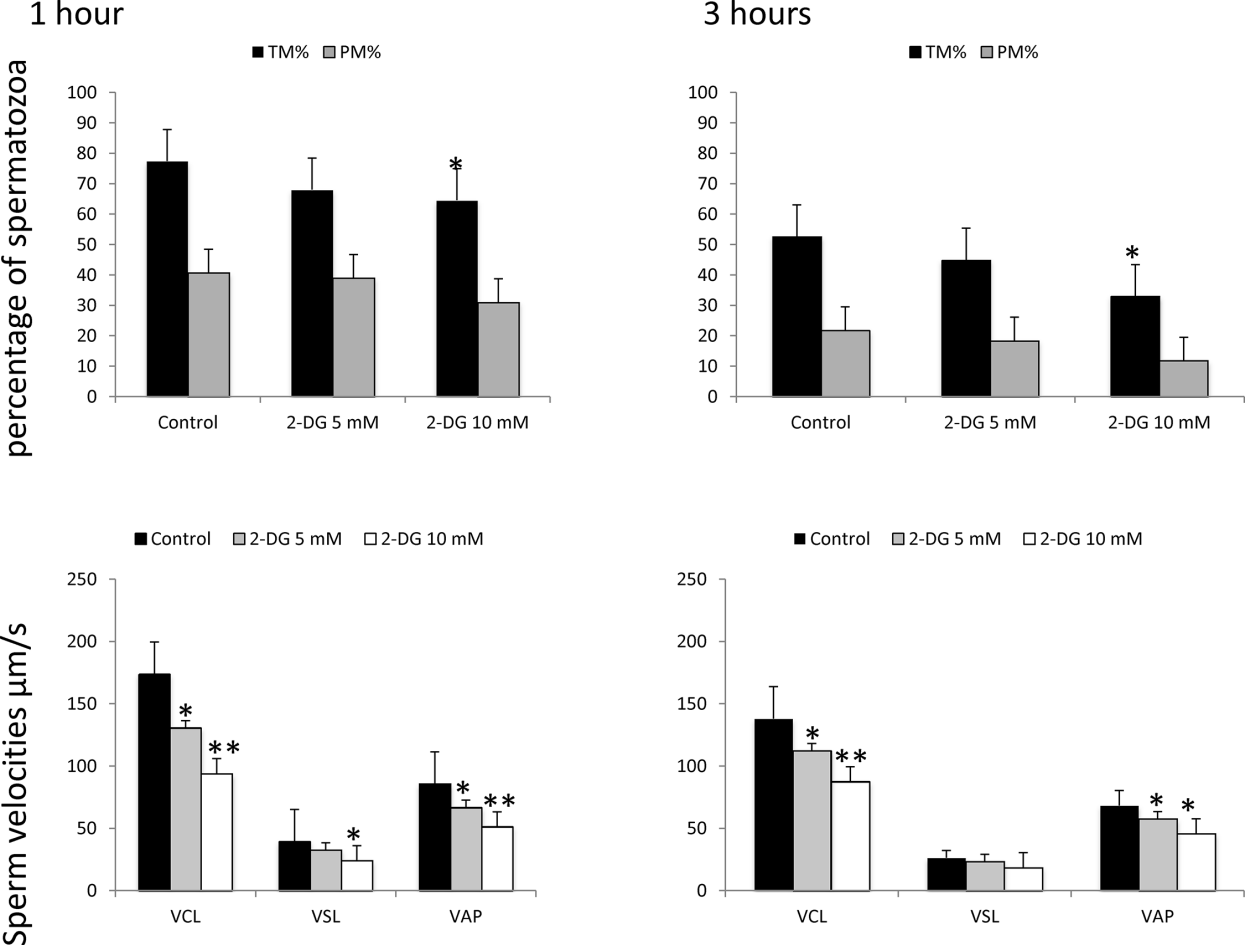
Effect of the inhibition of glycolysis on stallion sperm motility and kinematics. Stallion sperm were incubated in presence of 0, 5, and 10 mM of 2-deoxy-D-glucose as described in the materials and methods. The percentages of total motile sperm and progressively motile sperm and the sperm velocities were analyzed using a CASA system. *p<0.05, ** p<0.001. The results are given as the means ± SD.

**Fig 12:**
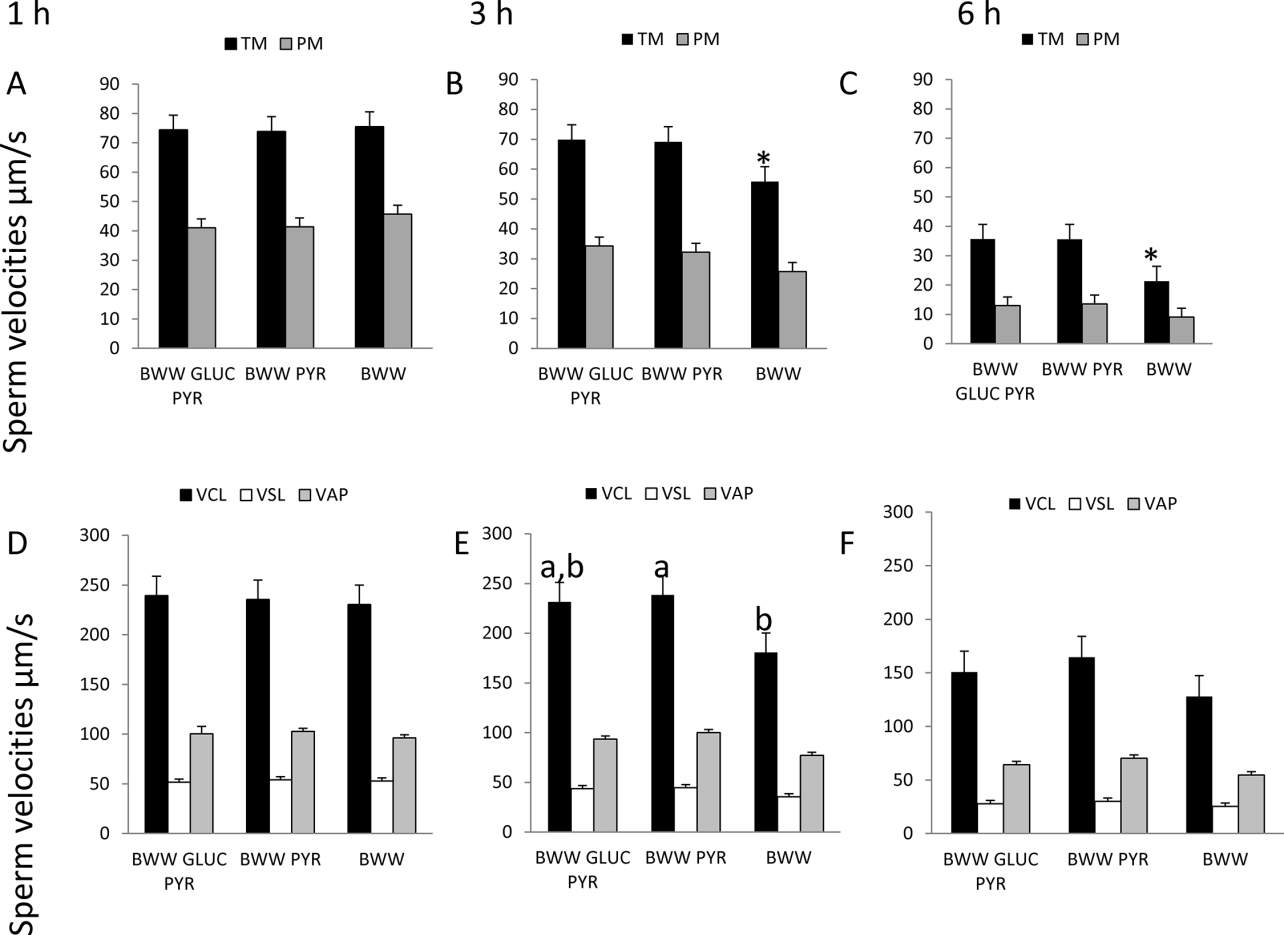
Effect of the incubation of stallion spermatozoa in BWW complete media (BWW GLUC PYR) devoid of glucose (BWW PYR) or devoid of glucose and pyruvate (BWW) on stallion sperm motility and kinematics. A, B and C, effects on the percentages of total motile (TM) and progressive motile (PM) after 1, 3 and 6 hours of incubation. D,E, F, effect on sperm velocities after 1, 3 and 6 hours of incubation. The percentages of total motile sperm and progressively motile sperm and the sperm velocities were analyzed using a CASA system. *p<0.05, in E bars with different superscripts represent significant differences (a, b p<0.05). The results are given as the means ± SD.

**Fig 13:**
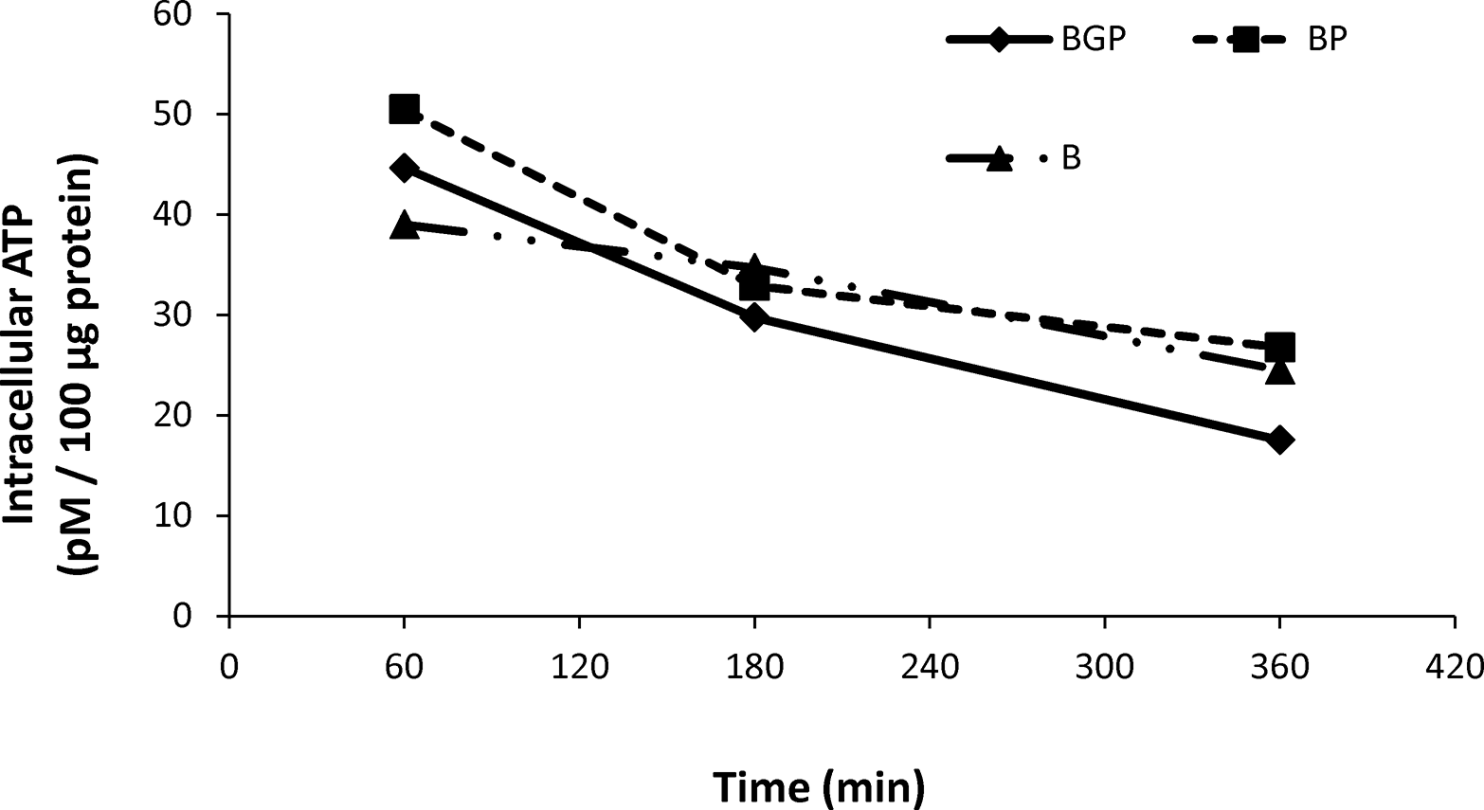
Effect of the incubation of stallion spermatozoa in BWW complete media (BWW GLUC PYR) devoid of glucose (BWW PYR) or devoid of glucose and pyruvate (BWW) on on the ATP content of stallion spermatozoa. ATP was determined as described in the materials and methods. The results are given as the means ± SD.

Because ATP production is not completely abolished and motility and membrane integrity are not completely suppressed, either membrane potential are not completely suppressed or other sources of ATP may be present. To test this hypothesis, stallion sperm were incubated in presence of 0, 5, and 10 mM of 2-Deoxy-D-Glucose. This is a glucose analog that inhibits glycolysis via its actions on hexokinase, the rate limiting step of glycolysis. It is phosphorylated by hexokinase to 2-DG-P and cannot be further metabolized by phosphoglucose isomerase. This leads to the accumulation of 2-DG-P in the cell and the depletion in cellular ATP. The inhibitor 2-deoxyglucose induced a 17% reduction in total motility after 1 hour of incubation and 45.8% after 1 hour of incubation (p<0.01) (Fig 11) without any effect on the percentage of progressive motile sperm, mitochondria or membrane permeability and integrity, the percentage of spermatozoa with intact membranes after 1 h of incubation was 54.1 in controls, 56.0, 56.7 and 55. 7 in samples incubated in the presence of 2, 5 and 10 mM of the inhibitor. After 3 h of incubation these percentages were 37 for controls, 31, 26 and 29 for samples supplemented with 2, 5 and 10 mM 2-DG. The percentages of spermatozoa with low mitochondrial membrane potential after 1 hour of incubation were 36, 36, 35 and 38 in controls and samples incubated in presence of 2, 5 and 10 mM of the analog. After 3 h of incubation these percentages were 47, 51, 51 and 54 in samples incubated in presence of 0, 2, 5, and 10 mM of the analog respectively. Additionally stallion sperm was incubated in a medium devoid of any kind of glucose, and in the absence of glucose and pyruvate. The only effect observed was a reduction in the percentage of total motile sperm after three hours of incubation in media devoid of pyruvate and glucose (Fig 12B and 12C, and a reduction in the VCL in media devoid of pyruvate and glucose compared to media devoid only of pyruvate (Fig 12E); this occurred only at three hours of incubation and this difference was not longer present after 6 hours. No changes were observed in ATP content (Fig 13).

### Relationship between reactive oxygen species and motility, membrane integrity, and mitochondrial membrane potential

Positive correlations were observed between different indicators of ROS production and sperm motility and velocity, with Mitosox-positive spermatozoa showing small but positive correlations with the percentage of total motile sperm (R^2^ = 0.219, p<0.05) and sperm velocities (VCL, R = 0.314, p<0.01; VAP R = 0.229, p<0.01) (Table 1). Similarly significant correlations were found between CellRox-positive spermatozoa and the percentages of total motile spermatozoa (R = 0,180, p<0.05); progressive motile spermatozoa (R = 0.251, p<0.01); and VCL, VSL and VAP (Table 2). Interestingly the percentage of CellRox-positive sperm correlated with the percentage of intact sperm (R = 0.501, p<0.01) and the percentage of spermatozoa with high mitochondrial membrane potential (R = 0.363, p<0.01); other correlations found are summarized in Table 3.

**Table 1:** Significant correlations between Mitosox-positivity and sperm motility and velocity.

	TM%	VCL μm/s	VAP μm/s	VSL μm/s
Mitosox +	R = 0.219*	R = 0.314**	R = 0.319**	R = 0.198*

Mitosox + spermatozoa showing mitochondrial production of superoxide radical, TM%—percentage of total motile sperm, VCL—curvilinear velocity, VAP—average path velocity, VSL—straight-line velocity.

*p<0.05

** p<0.01

**Table 2:** Significant correlations between different indicators of the production of ROS and parameters of sperm motility and velocities.

	TM%	PM%	VCL μm/s	VAP μm/s	VSL μm/s
CellRox (live cells)	R = 0.180*	R = 0.251**	R = 0.313**	R = 0.229**	R = 0.285**

*p<0.05

** p<0.01

CellRox (Live Cells) percentage of live spermatozoa showing ROS production, TM%—percentage of total motile sperm, VCL—curvilinear velocity, VAP—average path velocity, VSL—straight-line velocity.

**Table 3:** Significant correlations between different indicators of the production of ROS and membrane intactness and mitochondrial functionality.

	Intact sperm	YoPro+	JC-high
Mitosox+	R = 0.302**	R = 0.200**	R = 0.286**
H_2_DCFDA	R = 0.269**	R = 0.218**	R = 0.302**
CellRox (live cells)	R = 0.501**	n.s	R = 0.363**
HE	R = -0.348**	n.s	n.s.

** p<0.01, n.s non-significant

Mitosox+ spermatozoa show mitochondrial production of superoxide radical, H_2_DCFDA—percentage of spermatozoa showing hydrogen peroxide production, CellRox (Live Cells)—percentage of live spermatozoa showing ROS production, HE—percentage of spermatozoa showing superoxide production, Intact sperm—percentage of spermatozoa with completely intact membranes, YoPro+—percentage of spermatozoa with intact membranes but with increased permeability, JC- high percentage of spermatozoa with high mitochondrial membrane potential.

## Discussion

We studied the effects of interrupting the electron flux in the mitochondria of stallion sperm at two specific points, complex I (NADH-ubiquinone oxidoreductase), and complex III (co-enzyme Q-cytochrome c reductase). The major effects of these interventions were reductions in motility and velocities of the sperm. ATP content was also reduced, as were the mitochondrial membrane potential and membrane intactness and permeability. Intriguing effects were observed in terms of ROS production. The inhibition of complexes I and III reduced ROS production as assayed using the CellRox deep red reagent to measure oxidative stress. This is a paradoxical result because complexes I and III are recognized as major sources of ROS, both in somatic cells [18] and in spermatozoa [6]. This observation may be due to the fact that this probe is mainly sensitive to superoxide anion. According to the manufacturer the probe used in our experiment is sensitive to only the hydroxyl radical and the superoxide anion. Because significant amounts of divalent cations are unlikely in our model, the most likely explanation for our findings is that once the ETC is disrupted, the mitochondrial potential collapses and superoxide is no longer produced due to the interruption of electron transfer and subsequent electron leakage. Hydroethidine detected increased superoxide production in samples incubated in presence of antimycin 10 μM after 1 hour of incubation. However, the discrepancy between CellRox and hydrotheidine can be explained by the fact the CellRox was used to detect superoxide only in live cells, whereas hydroethidine was used in the whole population. The observed increase is therefore explained by superoxide produced by dead cells. This theory is also supported by the decrease in the percentage of intact sperm observed in the samples supplemented with antimycin and by the positive correlations found between CellRox-positivity and motility and membrane integrity

This hypothesis is also supported by the rapid collapse in mitochondrial membrane potential observed after the inhibition of both complexes, further suggesting that the interruption of the ETC reduces electron leakage. On the other hand, previous reports have demonstrated that mitochondrial inhibitors either increase or decrease ROS production [30]. In somatic cells, a small mitochondrial depolarization leads to increased ROS generation, whereas a more profound mitochondrial depolarization reduces ROS; this is consistent with the model where under resting conditions, 1% to 2% or O2 used in the ETC is not completely reduced, leading to the generation of superoxide anion [31].

Superoxide anion may be an indicator of intensely metabolically active spermatozoa [8]. Further evidence in favor of this assumption can be found in the observations that both inhibitors reduced ATP and that positive (but weak) correlations were found between Mitosox-positive cells and sperm motility and velocity. To further investigate the mitochondrial production of reactive oxygen species, a probe specific for the mitochondrial superoxide production was used. With this reagent, only the inhibition of complex I led to increased mitochondrial superoxide production after six hours of incubation; this was also accompanied by parallel increases in hydrogen peroxide. However the increase in mitosox positivity may also be due to an artifact, due to ethidium contamination of the probe, and this is a clear limitation of this assay[32]. Rotenone induces hydrogen peroxide in somatic cell’s mitochondria[18], and this was also evident in stallion spermatozoa.

Antimycin induced hydrogen peroxide production has been described in somatic cells [18]. Antimycin leads to the generation of the semiquinone radical [33], which then stabilizes by shedding its electrons to oxygen to create superoxide in the intramembranous space, dismutates to H_2_O_2_ under the influence of superoxide dismutase, and escapes to the outside of the cell. We were unable to detect increases in hydrogen peroxide, even after long incubation periods; this may be due to the presence of antioxidant enzymes in this space or reduced superoxide production.

Both inhibitors reduced ATP production in stallion spermatozoa accompanied by a significant reduction in sperm motility and velocity and by increases in membrane permeability. However, sperm motility and velocity were not abolished, To determine the potential role of glycolysis as a source of energy for motility, glycolysis was inhibited using a specific inhibitor, and also stallion sperm was incubated in media devoid of any kind of glucose and devoid of glucose and pyruvate. The inhibition of glycolysis resulted in dramatically decreased sperm velocities, and also induced changes in the percentage of progressive motile sperm with the higher dosage. However this approach has some caveats, the futile phosphorylation of 2-DG by hexokinase will result in ATP depletion, for this reason is difficult to determine whether decreased ATP production is due to depletion or reduced rate of ATP production. Incubation of stallion spermatozoa in media devoid of glucose and pyruvate had no effect on ATP, although there was an effect on motility and sperm velocities after three hours of incubation. These results suggest that although OXPHOS is the main source for sperm motility, glycolysis is necessary to support the sperm movement after long incubation periods.

Although previous reports indicate that stallion spermatozoa are highly dependent on mitochondrial ATP [8, 34], the role of glycolysis have not been investigated to date. Our results indicate that on one hand, stallion spermatozoa are highly dependent on mitochondrial ATP for motility, confirming recent findings[8, 34]. However, this study also demonstrated for the first time that glycolysis has a role supporting sperm movement.

Stallion spermatozoa have high concentration of antioxidant enzymes [35, 36], probably representing an evolutionary adaptation to their intense mitochondrial activity. Supporting this assumption are the positive correlations found between markers of oxidative stress and sperm function in the present study. Recent findings indicate a relationship between oxidative stress and field fertility in stallions [8]. The correlations found in our study, although significant, were weak and moderate. Previous studies have demonstrated that cryopreservation induces lipid peroxidation [14] and that antioxidants controversially improve the outcome of cryopreservation [37–39]. In other species, antioxidant supplementation has successfully [9, 40] improved the outcome of cryopreservation. Our understanding of the function of mitochondrial ROS in somatic cells has changed in recent years [41, 42], and ROSs are no longer believed to be simply a toxic byproduct of oxidative metabolism. On the contrary, they are considered to be important regulators of many cellular functions; ROS can cause reversible post-translational protein modifications to regulate signaling pathways. This role may be of special importance in spermatozoa; spermatozoa are translationally silent cells that are unable to synthetize de novo proteins and can only rely on post-translational modifications to regulate their functions[43].

In short, it can be concluded that stallion sperm mostly rely on OXPHOS to produce ATP for motility. The inhibition of complex I of the ETC in the mitochondria leads to reduced motility due decreased ATP and to increased hydrogen peroxide production. These findings have implications for the meaning and significance of reactive oxygen production by stallion spermatozoa and may influence strategies for sperm preservation.
